# SLC7A2 serves as a potential biomarker and therapeutic target for ovarian cancer

**DOI:** 10.18632/aging.103433

**Published:** 2020-07-09

**Authors:** Tianshui Sun, Fangfang Bi, Zhuonan Liu, Qing Yang

**Affiliations:** 1 Department of Obstetrics and Gynecology, Shengjing Hospital of China Medical University, Shenyang, China; 2 Department of Urology, First Hospital of China Medical University, Shenyang, China

**Keywords:** SLC7A2, solute carrier family, ovarian cancer, amino acid transporter, bioinformatics

## Abstract

The solute carrier (SLC) family is the largest group of membrane transporters, but their functions in ovarian cancer (OV) remain unclear. We analyzed SLC family members with amino acids-transporting functions in OV. The mRNA expression levels and prognostic values of SLCs in OV were analyzed in the Gene Expression Profiling Interactive Analysis and Kaplan–Meier Plotter database. Solute carrier family 7 member 2 (SLC7A2), which showed differential expression and the most significant prognostic value, was selected for further analyses. The cBioPortal database, Gene Set Enrichment Analysis and Weighted Correlation Network Analysis were used to explore the potential functions and molecular mechanisms of SLC7A2 in OV. Validations in our own samples and in Gene Expression Omnibus datasets were conducted. Functional validation in OV cell lines was carried out. In total, 73 SLC family members were analyzed. Seven members were upregulated while 11 members were downregulated in OV and 15 members were protective factors for prognosis while 12 members were risk factors. SLC7A2 was downregulated in OV, and it was positively associated with prognosis. Knockdown of SLC7A2 promoted viability, invasion and migration of OV cells. These SLC family members and in particular SLC7A2 represented novel biomarkers for diagnosis and treatment for OV.

## INTRODUCTION

Ovarian cancer has the highest mortality rate of all gynecological cancers [1]. Statistical analyses indicate that ovarian cancer caused 295,414 new cases and 184,799 cancer-associated deaths globally in 2018 [1, 2]. Due to lack of typical symptoms and reliable early detection methods, about 70% of ovarian cancer patients are diagnosed at an advanced stage [3]. Conventional treatment for ovarian cancer involves cytoreductive surgery followed by platinum-based chemotherapy [3]. However, 70% of patients with advanced ovarian cancer relapse within 1 to 2 years after treatment [4]. Because of tremendous threat ovarian cancer brings to women’s health, studies aimed at molecular mechanisms are in urgent need to identify effective tumor biomarkers for prognosis improvement by underlying tumorigenesis and progression of ovarian cancer.

The solute carrier (SLC) superfamily is the second largest family of membrane proteins and the largest group of transporters, which comprise more than 420 members from 65 families [5, 6]. Acting as cotransporters, uniporters or channels, SLCs mediate the movement of substrates across membranes based on electrochemical or ion gradients and transport a diverse range of substances, including ions, drugs, and metabolites [6, 7]. SLC dysfunction can lead to a variety of diseases, such as diabetes, Alzheimer's disease, and heart disease [8–10]. Moreover, emerging evidence suggests that SLCs are involved in the genesis and progression of various cancers, including bladder urothelial carcinoma, colorectal cancer, and breast cancer [11–13]. However, few studies have been demonstrated the role SLCs paly in ovarian cancer and thus it’s worth further explorations. As important nutrients in human body, various amino acids like glutamine, asparagine, serine, and arginine have exhibited association with tumorigenesis and prognosis of cancers, including ovarian cancer [14, 15]. In terms of SLC families, amino acid transport is mediated by members of the SLC1, SLC3/7, SLC6, SLC25, SLC32, SLC36, SLC38, and SLC43 families [16]. However, their functions in ovarian cancer remain unclear.

Identification of new cancer biomarkers has become easier and more accurate with the advent of large available databases and web-based tools based on RNA-sequencing techniques. Here we focused on members of the above-mentioned SLC family members with amino acid-transporting functions. Various databases, including the Gene Expression Profiling Interactive Analysis (GEPIA) databases [17], the Kaplan–Meier Plotter database [18], the c-Bioportal database [19], the Cancer Cell Line Encyclopedia (CCLE) database [20], The Cancer Genome Atlas (TCGA) database [21] and the Gene Expression Omnibus (GEO) database were utilized for analyses of SLCs in ovarian cancer, combined with clinical sample validation and functional experiments. Our study was aimed at exploring amino acid-transporting SLC family members in ovarian cancer and attempted to provide a theoretical basis for improving the diagnosis, therapy, and prognosis of ovarian cancer.

## RESULTS

### Identification of differentially expressed SLC family members

**Figure 1:**
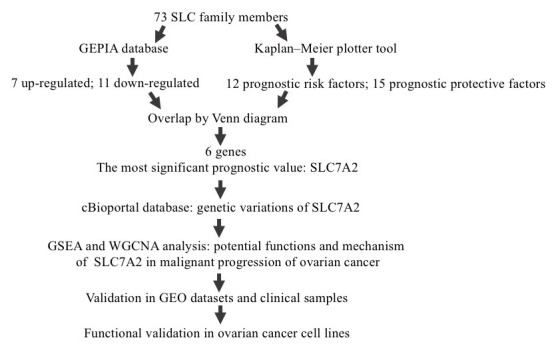
**Study flow diagram.** SLC, solute carrier; GEPIA, Gene Expression Profiling Interactive Analysis; GO, Gene Ontology; SLC7A2, solute carrier family 7 member 2; GSEA, Gene Set Enrichment Analysis; WGCNA, Weighted Correlation Network Analysis; GEO, Gene Expression Omnibus.

**Figure 2:**
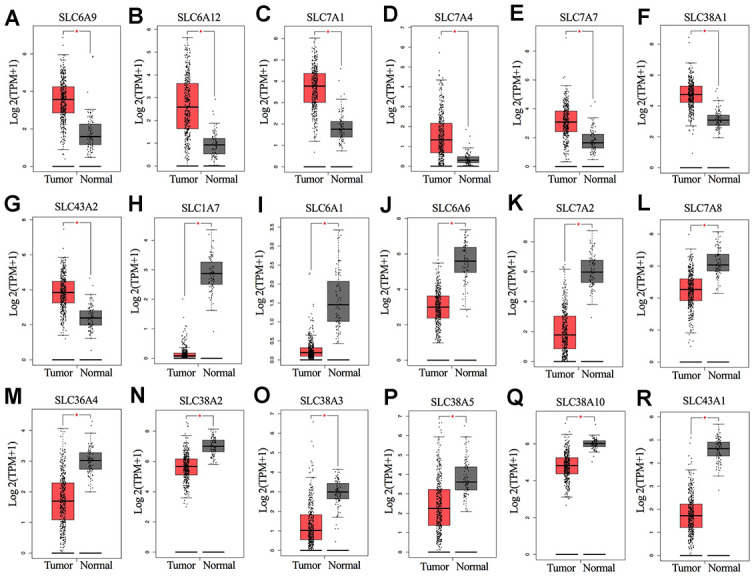
****(**A**–**R**) mRNA expression levels of SLC family members in ovarian cancer (426 samples) and normal ovarian tissues (88 samples) (GEPIA). *P < 0.01. SLC, solute carrier.

The overall design of this study is shown in Figure 1. We compared the mRNA expression levels of the 73 SLC family members between ovarian cancers and normal ovarian tissues in the GEPIA database (Supplementary File 1). Eighteen SLC family members were identified to be significantly differentially expressed (p<0.01) (Figure 2A–2R), among which seven members were significantly upregulated (SLC6A9, SLC6A12, SLC7A1, SLC7A4, SLC7A7, SLC38A1, and SLC43A2) while eleven members were downregulated (SLC1A7, SLC6A1, SLC6A6, SLC7A2, SLC7A8, SLC36A4, SLC38A2, SLC38A3, SLC38A5, SLC38A10 and SLC43A1).

### Prognostic value of SLC family members

**Figure 3:**
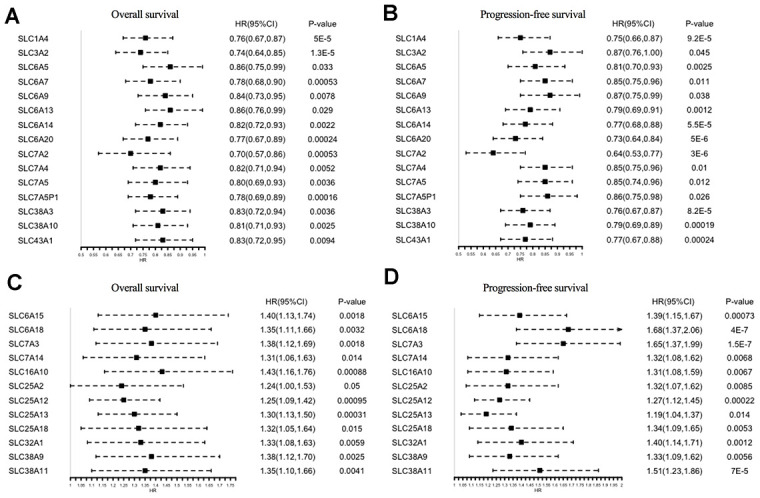
**Prognostic values of SLC family members in ovarian cancer (Kaplan–Meier Plotter).** (**A**, **B**) HRs of prognostic protective SLC family members in ovarian cancer. (**C**, **D**) HRs of prognostic risk SLC family members in ovarian cancer. SLC, solute carrier; HR, hazard ratio; CI, confidence interval.

The prognostic significance of the 73 SLC family members in ovarian cancer patients was investigated using the Kaplan–Meier Plotter tool. In total, 27 genes were found to be significantly associated with both OS and progression-free survival (PFS) (Figure 3A–3D): 15 of them were protective factors for prognosis (HR < 1), whereas 12 were risk factors for prognosis (HR > 1).

**Figure 4:**
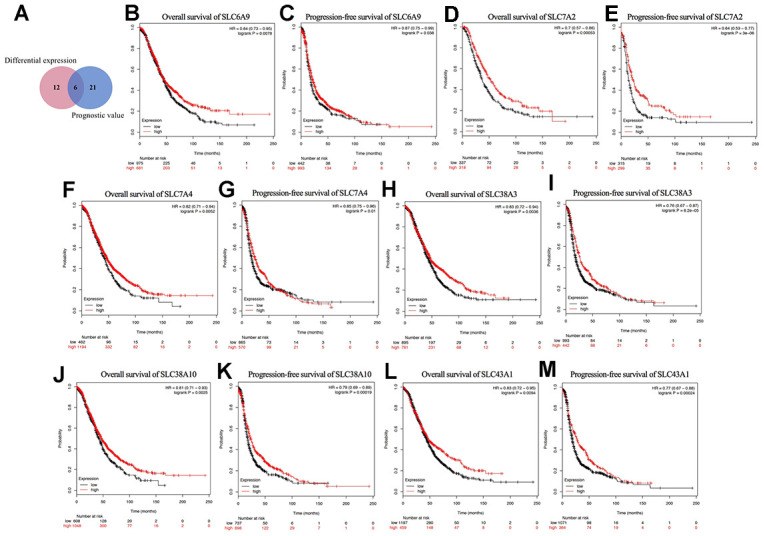
**Prognostic values of differential expressed SLC family members in ovarian cancer (Kaplan–Meier Plotter).** (**A**) Venn diagram for the screening of the genes with both significant differential expression and prognostic value. (**B**–**M**) Prognostic significance of individual SLC family members with both significant differential expression and prognostic value in ovarian cancer. SLC, solute carrier.

As shown in Figure 4A, a Venn diagram was generated to explore the intersection of the genes with significant differential expression and the genes with prognostic value, where six genes were identified: SLC6A9, SLC7A2, SLC7A4, SLC38A3, SLC38A10, and SLC43A1. Their Kaplan-Meier plots for OS and PFS are shown in Figure 4B–4M. Among the six genes, SLC7A2 showed the most significant prognostic value for both OS and PFS. The median OS values were 52.43 and 36.57 months and the median PFS values were 22.41 and 14 months for the high and low SLC7A2 expression groups, respectively. Therefore, we identified SLC7A2 as a marker associated with survival in ovarian cancer and focused on SLC7A2 in further exploration.

### Genetic variations of SLC7A2

**Figure 5:**
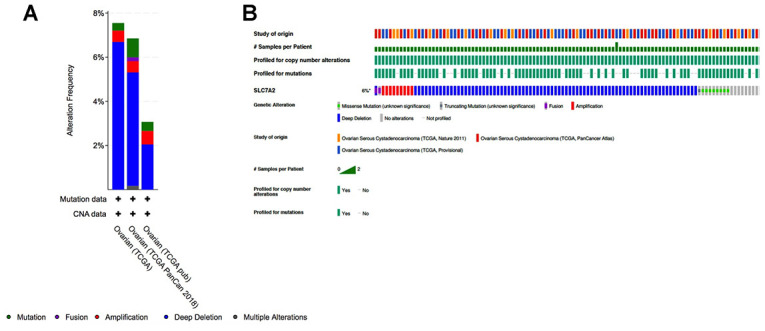
**Analyses of genetic variations in SLC7A2 in ovarian cancer (cBioPortal).** (**A**) Genetic variations in the SLC7A2 gene reported in different studies. (**B**) OncoPrint overview of the genetic variations in the SLC7A2 gene. TCGA, The Cancer Genome Atlas.

To determine if downregulation of SLC7A2 in ovarian cancer tissues were caused by genetic variations, we assessed genetic variations of SLC7A2 in the cBio-Portal database which contained information on 1680 ovarian cancer cases from three studies (TCGA, Nature 2011; TCGA, PanCancer Atlas; TCGA, Provisional). In this database, genetic variations of SLC7A2 showed incidence rates of 7.55% in TCGA, 6.85% in TCGA PanCancer Atlas, and 3.07% in TCGA Provisional (Figure 5). Deep deletion was the most common type, comprising more than half of all genetic variations (6.69% in TCGA; 5.14% in TCGA PanCancer Atlas; and 2.04% in TCGA Provisional). Mutation and amplification were the next most common types of genetic variations. Therefore, deep deletion might be one of the main mechanisms by which SLC7A2 is under-expressed in ovarian cancer.

### GSEA and WGCNA analysis

**Figure 6:**
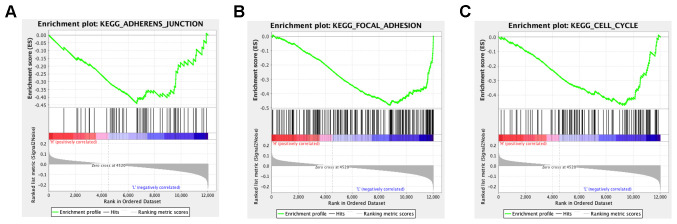
**GSEA analysis of SLC7A2-related enrichment gene sets.** (**A**) KEGG_ADHERENS_JUNCTION (P-value = 0.026; FDR = 0.237; Enrichment score = -0.440). (**B**) KEGG_FOCAL_ADHESION (P-value = 0.000; FDR = 0.096; Enrichment score = -0.482). (**C**) KEGG_CELL_CYCLE (P-value = 0.000; FDR = 0.117; Enrichment score = -0.470). GSEA: Gene set enrichment analysis; KEGG: Kyoto Encyclopedia of Genes and Genomes; FDR: False Discovery Rate.

**Figure 7:**
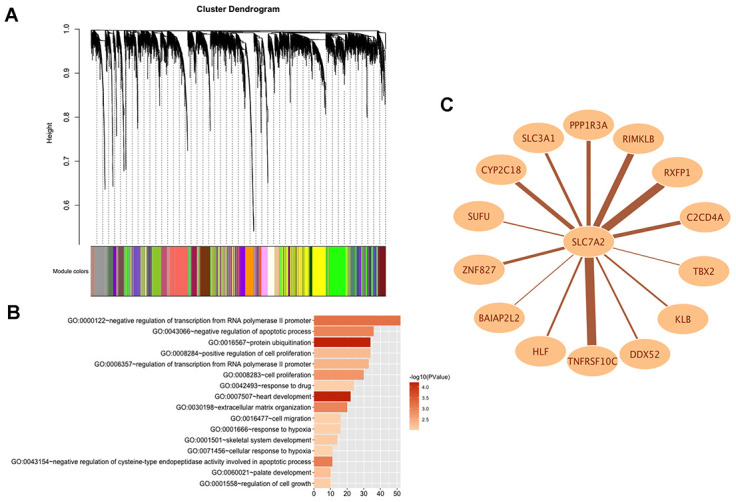
**WGCNA analysis of SLC7A2 in transcriptomic data of 44 ovarian cancer cell lines.** (**A**) Gene modules divided by WGCNA. (**B**) GO bioinformatics analysis of genes in the brown module. (**C**) Network of SLC7A2 and its connected genes. WGCNA, Weighted Correlation Network Analysis; GO, Gene Ontology.

In order to explore the potential functions and molecular mechanisms of SLC7A2 in ovarian cancer progression, we conducted Gene Set Enrichment Analysis (GSEA) in ovarian cancer tissues from TCGA database, which indicated enrichments of gene sets including adherens junction, focal adhesion and cell cycle in the samples with low SLC7A2 expression (Figure 6). We further analyzed the transcriptomic data of 44 ovarian cancer cell lines in CCLE database by Weighted Correlation Network Analysis (WGCNA). We used 20,486 genes to construct 88 modules (Figure 7A). The genes enriched in these modules were listed in Supplementary File 2. 943 genes, including SLC7A2, were enriched in the brown module. Functional enrichment analysis was carried out for the 943 genes and we found these genes were significantly associated with several processes that have been previously reported in tumorigenesis and progression of ovarian cancer, such as ‘protein ubiquitination’, ‘negative regulation of transcription from RNA polymerase II promoter’, ‘negative regulation of apoptotic process’ and ‘cell proliferation’ [22–24]. The terms with p-value < 0.01 and counts > 10 was shown in Figure 7B. What’s more, fourteen genes in the brown module were found connected with SLC7A2 and were visualized in network in Figure 7C.

### Validation of SLC7A2 in independent cohorts

**Figure 8:**
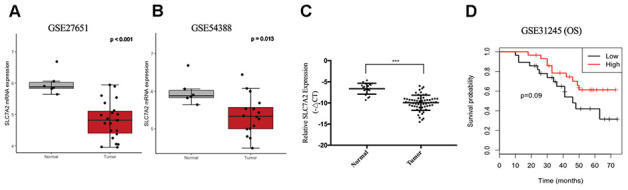
**Validation of SLC7A2 based on GEO datasets and clinical samples.** mRNA expression levels of SLC7A2 based on (**A**) GSE27651 (22 high-grade serous ovarian carcinomas and 6 normal ovarian tissues), (**B**) GSE54388 (16 epithelial ovarian cancer and 6 normal ovarian tissues) and (**C**) our clinical samples (60 ovarian cancer and 20 normal ovarian tissues). (**D**) Prognostic significance of SLC7A2 in ovarian cancer based on GSE31245 (58 ovarian cancer cases). ***P < 0.001. GEO, Gene Expression Omnibus.

For further validation of expression variation of SLC7A2, we compared its expression levels between ovarian cancer and normal ovarian tissues in the GSE27651 and GSE54388 datasets, respectively. SLC7A2 was downregulated in ovarian cancer in both datasets (Figure 8A, 8B). Furthermore, we examined the mRNA expression levels of SLC7A2 in our 60 primary ovarian cancer specimen and 20 normal ovarian tissues. qRT-PCR result showed that the expression levels of SLC7A2 were significantly lower in cancer tissues than in normal ovarian tissues (Figure 8C). Sixty patients with epithelial ovarian cancer were divided into high SLC7A2-expression groups and low SLC7A2-expression groups with the median -ΔCT as cutoff value. Statistical analyses revealed that patients ≥ 60 years old had significantly higher SLC7A2 expression than patients < 60 years old (p-value = 0.037). However, the SLC7A2 expression level did not significantly correlate with International Federation of Gynecology and Obstetrics (FIGO) stage, grade and pathologic type (Table 1).

**Table 1:** Relationships between SLC7A2 expression in epithelial ovarian cancer and clinicopathological parameters.

**Characteristic**	**n**	**Low**	**High**	**P value**
Age (years)				0.037
<60	26	17	9	
≥60	34	13	21	
Stage				0.64
FIGO III	55	27	28	
FIGO IV	5	3	2	
Grade				0.212
Well	2	0	2	
Moderate	21	9	12	
Poor	37	21	16	
Pathologic type				0.276
Serous	52	28	24	
Mucinous	1	0	1	
Endometrioid	1	0	1	
Clear cell carcinoma	5	1	4	
Undifferentiated adenocarcinoma	1	1	0	

FIGO, International Federation of Gynecology and Obstetrics.

To validate the prognostic value of SLC7A2 in ovarian cancer, we divided 58 patients in the GSE31245 datasets into two groups using the median mRNA expression level of SLC7A2 as a cutoff point. Compared with the low expression group, the high expression group showed better OS (Figure 8D).

### Functional validation of SLC7A2

**Figure 9:**
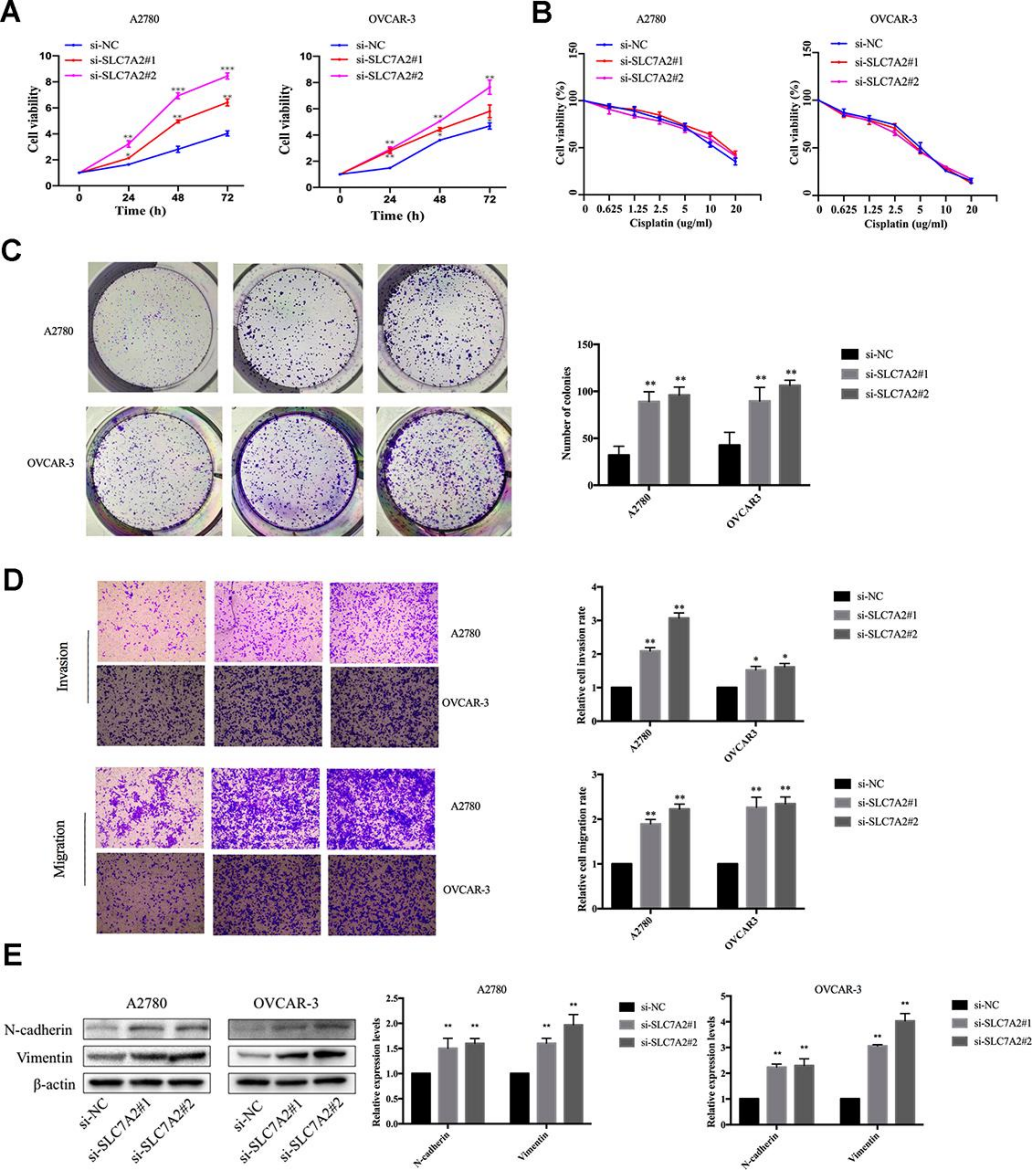
**Effects of SLC7A2 knockdown on the functions of ovarian cancer cell lines.** (**A**) Cell viability increased after SLC7A2 knockdown in the A2780 and OVCAR-3 cell lines. (**B**) Cell viability had no significant change after SLC7A2 knockdown in the A2780 and OVCAR-3 cell lines cultured in different concentrations of cisplatin for 48 h. (**C**) Number of colony increased after SLC7A2 knockdown in the A2780 and OVCAR-3 cell lines. (**D**) Cell invasion and migration increased after SLC7A2 knockdown in the A2780 and OVCAR-3 cell lines. (**E**) N-cadherin and Vimentin proteins of A2780 and OVCAR-3 cell lines increased after SLC7A2 knockdown. The data are shown as the mean ± SD (n=3). *p < 0.05, **p < 0.01, ***p < 0.001.

We performed functional experiments after knockdown of SLC7A2 by siRNAs in the A2780 and OVCAR-3 cell lines to validate the function of SLC7A2 in ovarian cancer. We used reverse transcription quantitative polymerase chain reaction (qRT-PCR) to examine the efficiency of knockdown (Supplementary Figure 1). The Cell Counting Kit-8 (CCK-8) experiments showed that an SLC7A2 decrease augmented cell viability (Figure 9A). Cisplatin resistance was one of the causes of ovarian cancer recurrence [4], so we explored whether SLC7A2 is related to ovarian cancer's reactivity to cisplatin. SLC7A2 knockdown had no effect on the sensitivity of ovarian cancer cells to cisplatin treatment (Figure 9B). We found that knockdown of SLC7A2 promoted colony formation (Figure 9C). The Transwell experiments showed increased invasion and migration ability of ovarian cancer cells after SLC7A2 knockdown (Figure 9D). The protein levels of markers of epithelial-mesenchymal transition, including N-cadherin and vimentin, showed consistent changes with the Transwell results (Figure 9E).

## DISCUSSION

As the second largest family of membrane proteins and the largest group of transporters, the SLC superfamily transports various substances [5, 6]. SLC family members have attracted increased attention in recent years, and their functions in cancer pathogenesis and progression were reported in previous studies [25, 26]. Nonetheless, their functions in ovarian cancer have rarely been studied and remains unclear. In this study, we first focused on 73 SLC family members involved in amino acid transport and investigated their expression and prognostic value in ovarian cancer. SLC6A12 were found to be highly expressed in ovarian cancer. Consistently, SLC6A12 shows upregulated expression in metastatic sites of ovarian cancer and has been proven to promote the invasion and migration of ovarian cancer cells in vitro [25]. In our study, the expression levels of SLC6A1 and SLC7A8 were found to be decreased in ovarian cancer. However, a previous study indicated that SLC6A1 silencing reduced ovarian cancer cell proliferation, migration, and invasion [27]. SLC6A1 was found to be upregulated while SLC7A8 was found to be downregulated in drug-resistant ovarian cancer cell lines [28]. SLC3A2, which was identified by us as a protective factor for ovarian cancer prognosis, was also reported as a protective factor for ovarian cancer cisplatin sensitivity [29]. These findings suggest that SLC family members play important roles in the tumorigenesis and progression of ovarian cancer but the functions and molecular mechanisms remain to be experimentally elucidated.

Our study further focused on SLC7A2, which had both significant differential expression and the most significant prognostic value. Studies had found that the expression levels of SLC7A2 were correlated with a survival advantage in patients with breast cancer [30]. Loss of SLC7A2 could exacerbate inflammation-associated colon tumorigenesis [31]. SLC7A2 was found expressing at relatively high levels in the skeletal muscle, placenta and ovary [32]. However, the functions of SLC7A2 in ovarian cancer pathogenesis and progression have not been explored. Our study indicated that SLC7A2 was downregulated in ovarian cancer and identified SLC7A2 as a marker associated with protective prognosis in ovarian cancer. Moreover, we observed in our patients that expression level of SLC7A2 was lower in younger patients, while it was not significantly correlated with FIGO stage, grade or pathologic type. Through analysis in the cBioportal database, we identified deep deletion as one of the main mechanisms by which SLC7A2 is under-expressed in ovarian cancer.

According to previous studies, SLC7A2 locates at cell membrane [33] and it is involved in the transport of cationic amino acids, including lysine, arginine, and ornithine [32]. Studies have found that single amino acid arginine deprivation triggers profound pro-survival autophagic response in human ovarian cancer SKOV3 cells [34]. Thus, we speculated arginine deprivation might be a potential mechanism via which down-regulated SLC7A2 promoted ovarian cancer viability. To detect more potential mechanisms of SLC7A2 in ovarian cancer progression, we conducted GSEA analysis in ovarian cancer tissue from TCGA database and found that under-expressed SLC7A2 might promote ovarian cancer progression by promoting adhesion and proliferation of ovarian cancer cells. We then performed WGCNA analysis with the transcriptomic data of 44 ovarian cancer cell lines in CCLE database. WGCNA analysis can cluster highly interconnected genes in the same gene module [35]. The result showed 943 genes including SLC7A2 were enriched in the brown module, which were proved to be associated with proliferation, apoptosis, adhesion and migration of cancer cells by functional enrichment analysis. Functional experiments provided validation in two ovarian cancer cell lines. The GO terms in Figure 7B could guide new investigations into understanding the mechanisms of SLC7A2 in progression of ovarian cancer. Moreover, 14 genes in brown module were found connected with SLC7A2 (Figure 7C), among which TNFRSF10C had the highest weight of connection with SLC7A2. Study reported that TNFRSF10C could protect cells against TRAIL mediated apoptosis by competing with TRAIL-R1 and R2 for binding to the ligand [36]. RXFP1 was a receptor for relaxins, which was a short circulating peptide hormone and was found to be upregulated in patients with epithelial ovarian cancer [37]. RXFP1 activation was reported to mediate anti-apoptotic functions, angiogenesis and cell invasion in cancer [38]. SLC3A1 was associated with cysteine uptake, increased level of reductive glutathione and protected cancer cells from reactive oxygen species [39]. SUFU was a negative regulator in the hedgehog signaling pathway [40]. High TBX2 expression was found to be associated with platinum-resistance of ovarian serous carcinoma [41]. Therefore, SLC7A2 might promote ovarian cancer progression by interacting directly or indirectly with these molecules. The detailed mechanisms still require further exploration.

Our study revealed the abnormal expressions and prognostic values of amino acid transporter SLC family members in ovarian cancer. In addition, we highlighted the association between SLC7A2 and viability, invasion, migration of ovarian cancer cells. SLCs might be novel biomarkers for the diagnosis, therapy, and prognostic prediction for patients with ovarian cancer. More importance should be attached to SLC7A2 and other SLC family members in further studies of ovarian cancer and further explorations into relative molecular mechanisms are worth performing.

## MATERIALS AND METHODS

### Bioinformatic analysis

We obtained genes in SLC families form the amino acid transport gene set (GO_AMINO_ACID_TRANSPORT, M13394) in the Molecular Signatures Database v7.0 (http://software.broadinstitute.org/gsea/msigdb/genesets.jsp). We analyzed the mRNA expression levels of these genes in ovarian cancer and normal ovarian tissues in the GEPIA database (http://gepia.cancer-pku.cn/) [17]. The prognostic values of these SLC family members were assessed with the Kaplan–Meier Plotter tool (http://kmplot.com) [18]. Hazard ratios (HRs), 95% confidence intervals (CIs), and log-rank P-values were compared between high and low expression groups. Genetic variations were evaluated in the cBioPortal database (http://www.cbioportal.org) [19].

Gene expression RNA-seq data of TCGA ovarian serous cystadenocarcinoma (OV) was downloaded from the University of California Santa Cruz (UCSC) Xena databases (https://tcga.xenahubs.net). GSEA software (version 4.3.0; https://www.gsea-msigdb.org/gsea/index.jsp) was used for GSEA analysis. The gene sets database was c2.cp.kegg.v7.1.symbols.gmt. Enrichment analysis was performed on the expression spectrum data and attribute files after high and low grouping using the default-weighted enrichment analysis method. The random assortment frequency was set as 1,000.

The transcriptomic data of 44 ovarian cancer cell lines in CCLE project were obtained from the GEO database (accession numbers: GSE36133) (https://www.ncbi.nlm.nih.gov/geo/), uniformly processed, and normalized using the Robust Multichip Average algorithm with the Bioconductor package “affy” in the R programming language (version 3.5.2; http://www.r-project.org). All genes were involved to perform the WGCNA analysis by using “WGCNA” package in R software. The parameters were set as follows: minModuleSize = 30, and mergeCutHeight = 0.25. Then, gene network was exported to and visualized with Cytoscape (version 3.7.1; https://cytoscape.org). The Functional Annotation Result Summary tool from the Database for Annotation, Visualization, and Integrated Discovery (DAVID version 6.8; https://david.ncifcrf.gov/tools.jsp) was used for Gene Ontology Biological Process analysis [42].

The raw data (.CEL files) of three datasets were obtained from the GEO database (accession numbers: GSE27651, GSE54388, and GSE31245). GSE27651 and GSE54388 were used to validate the differential expression of SLC7A2 in ovarian cancer and normal ovarian tissues. GSE31245 was used to validate the prognostic value of SLC7A2 in ovarian cancer. The R packages “survival” and “survminer” were used for prognostic analysis and for building Kaplan-Meier plots of overall survival (OS).

### Clinical samples

Sixty ovarian primary cancer and 20 normal ovarian tissues were obtained from patients who underwent resection in Shengjing Hospital of China Medical University from 2016 to 2018. The study was approved by the Research Ethics Committee of China Medical University, and all patients signed written informed consent.

### Cell lines and transfection

A2780 and OVCAR-3 cells were purchased from the Chinese Academy of Sciences Cell Bank (Shanghai, China). All cells were cultured at 37 °C in a 5% CO2 atmosphere and were maintained in RPMI 1640 medium (Bioind, Kibbutz Beit Haemek, Israel), supplemented with 10% fetal bovine serum (FBS; Bioind, Kibbutz Beit Haemek, Israel).

Small interfering RNAs (siRNAs) targeting SLC7A2 were ordered from GenePharma (Suzhou, China). Lipofectamine™ 3000 Transfection Reagent (Invitrogen, Carlsbad, USA) was used for transfection. Cells in 6-well plates were transfected with control siRNA: forward UUCUCCGAACGUGUCACGUTT, reverse ACGUGACACGUUCGGAGAATT; SLC7A2 siRNA#1: forward CCAAAUUAUGCCGCUGCUUTT, reverse AAGCAGCGGC AUAAUUUGGTT; or SLC7A2 siRNA#2: forward GGACAUACUUCAGAAUGAATT, reverse UUCAUUCUGAAGUAUGUCCTT.

### Reverse transcription quantitative polymerase chain reaction

Total tissue RNA was extracted using the RNAiso Plus reagent (Takara Bio, Kusatsu, Japan). RNA concentration and purity were assessed with a Nano Drop 2000 system (Thermo Fisher, Carlsbad, USA). Total RNA was reverse transcribed using the PrimeScript™ RT reagent Kit with gDNA Eraser (Perfect Real Time) (Takara Bio, Kusatsu, Japan). Real-time quantitative polymerase chain reaction (qPCR) was performed using SYBR® Premix Ex Taq™ II (Tli RNaseH Plus) (Takara Bio, Kusatsu, Japan). The primer sequences were as follows: forward, GGCGTTGGAAGCACCCTTGG; reverse, GGCAGCAATGGGAAGGACAC. The PCR reactions were performed on an ABI 7500 Fast system (Life Technologies, Carlsbad, USA). Gene expression was calculated relative to that of ACTB using the 2^−ΔΔCt^ method.

### Colony formation assay

Cells were transfected with siRNAs for 24 h. They were plated into 6-well plates (200 cells/well), cultured for 2 weeks, and fixed with 4% paraformaldehyde and stained with 0.5% crystal violet. Colonies (> 50 cells) were manually counted.

### Cell viability assay

Cell viability was evaluated using a CCK-8 (Bimake, Houston, USA). Cells were transfected with siRNAs for 24 h. Cell suspension was inoculated in 96-well plates (3×10^3^cells/well). CCK-8 solution (10 μl) was added to the wells every 24 h and the cells were incubated for 1 h. The absorbance was measured at 450 nm using a microplate reader.

In cisplatin reactivity experiments, cells were transfected with siRNAs for 24 h and were inoculated in 96-well plates (8×10^3^cells/well). The cells were treated with cisplatin (Sigma, Saint Louis, USA) at various concentrations for 48 h. CCK-8 solution (10 μl) was added to each well, and the cells were incubated for 2 h. The absorbance was measured at 450 nm using a microplate reader.

### Cell invasion and migration assays

Cells were transfected with siRNAs for 24 h. Matrigel invasion assays were performed in 24-well plates using Transwell polycarbonate filters with an 8.0-μm pore size (Corning, New York, USA). 600 μl medium containing 10% FBS was added to the lower chamber. Cells in serum-free medium (2×10^4^cells/200 μl) were seeded into the upper chambers pre-coated with Matrigel (BD Biosciences, San Jose, USA). After incubation at 37°C for 24 h, the cells were fixed with 4% paraformaldehyde and stained with 0.5% crystal violet. The invaded cells were photographed and counted. The migration assay was similar but no Matrigel was added to the upper membrane.

### Western blot analysis

Total cell proteins were extracted using RIPA lysate (Beyotime, Shanghai, China). Proteins were separated using 10% SDS-PAGE and transferred onto PVDF membranes (Millipore, Massachusetts, USA). After being blocked at room temperature for 2 h, the membranes were incubated with primary anti-N-cadherin (1:2000; Proteintech, Chicago, USA), anti-vimentin (1:10000; Proteintech, Chicago, USA) or anti-β-actin (1:5000; Proteintech, Chicago, USA) antibodies. The membranes were then washed and incubated with secondary antibodies. Protein bands were detected with enhanced chemiluminescence (Thermo Scientific, Carlsbad, USA).

### Statistical analysis

Data are expressed as the mean ± standard deviation of at least three independent experiments. Statistical analyses were performed with GraphPad Prism 7.0 and the R programming language version 3.5.2. The count data were analyzed by Chi squared tests. The measurement data were analyzed using t-test. Survival analysis was performed with the Kaplan-Meier method and the log-rank test. P < 0.05 was considered statistically significant unless stated otherwise.

## Supplementary Material

Supplementary Figure 1

Supplementary File 1

Supplementary File 2
