# Supplementary figures and images for: SLC7A2 serves as a potential biomarker and therapeutic target for ovarian cancer
Source: Aging (Albany NY). 2020 Jul 9;12(13):13281–96. doi: 10.18632/aging.103433 (PMC7377849; doi:10.18632/aging.103433)

## SUPPLEMENTARY FIGURE

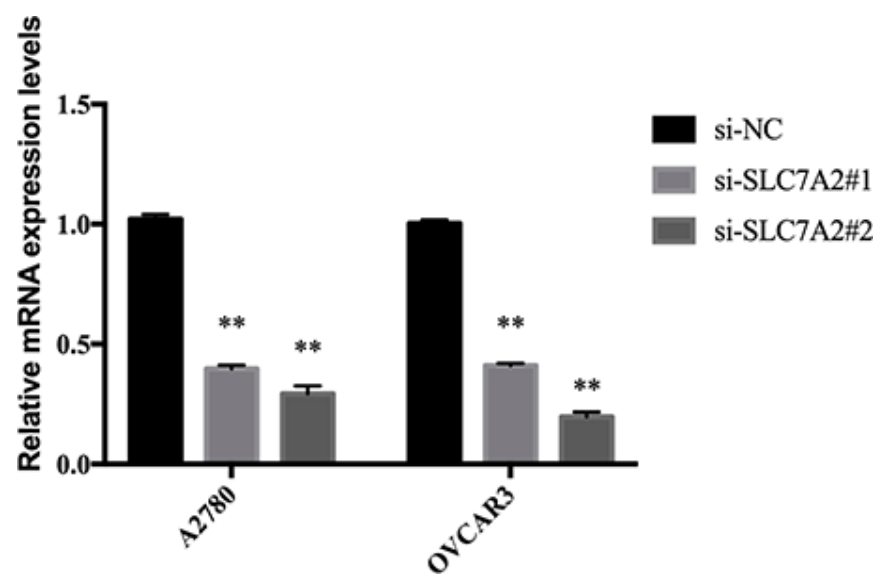

Supplementary Figure 1. The knockdown efficiency of the si-SLC7A2.

Supplement: Supplementary Figure 1 [file aging-12-103433-s002..pdf]
